# CYP3A5*3 and *SLCO1B1* c.521T>C Polymorphisms Influence the Pharmacokinetics of Atorvastatin and 2-Hydroxy Atorvastatin

**DOI:** 10.3390/pharmaceutics14071491

**Published:** 2022-07-18

**Authors:** Jin-Woo Park, Jong-Min Kim, Hwa-Young Lee, Jihyeon Noh, Kyoung-Ah Kim, Ji-Young Park

**Affiliations:** 1 Department of Clinical Pharmacology and Toxicology, Korea University Anam Hospital, Korea University College of Medicine, Seoul 02841, Korea; parkzinu@korea.ac.kr (J.-W.P.); jmk157@korea.ac.kr (J.-M.K.); hylee5973@gmail.com (H.-Y.L.); njh2535@korea.ac.kr (J.N.); kakim920@kumc.or.kr (K.-A.K.); 2 Department of Neurology, Korea University Medical Center, Seoul 02841, Korea; 3 Division of Clinical Pharmacology, Vanderbilt University School of Medicine, Nashville, TN 37232, USA

**Keywords:** atorvastatin, 2-hydroxy atorvastatin, *SLCO1B1*, *CYP3A5*, SNP

## Abstract

There is a large variability in individual responses to atorvastatin administration. This study assessed the pharmacogenetic effects of solute carrier organic anion transporter family member 1B1 (*SLCO1B1*, c.388A>G and c.521T>C) and cytochrome P450 3A5 (*CYP3A5*, CYP3A5*3) genetic polymorphisms on the pharmacokinetics of atorvastatin and its active metabolite, 2-hydroxy (2-OH) atorvastatin, in 46 individuals who were administered a clinically used single oral dosage of 80 mg. The C_max_ and AUC of atorvastatin in CYP3A5*3/*3 carriers were 2.6- and 2.8-fold higher, respectively, than those in CYP3A5*1/*1 carriers, and similar results were observed for 2-OH atorvastatin pharmacokinetics. *SLCO1B1* c.521T>C also increased the AUC of atorvastatin and 2-OH atorvastatin. The AUC ratio of atorvastatin and 2-OH atorvastatin were not affected by *SLCO1B1* c.388A>G or c.521T>C, whereas CYP3A5*3 reduced the AUC ratio. In an analysis evaluating the simultaneous effect of the *SLCO1B1* c.521T>C and CYP3A5*3 polymorphisms, *SLCO1B1* c.521TT/CYP3A5*1/*1 carriers showed lower C_max_ and AUC values for atorvastatin and 2-OH atorvastatin than in individuals with the *SLCO1B1* c.521T>C and/or CYP3A5*3 genotypes. Among the participants with the *SLCO1B1* c.521TT genotype, the CYP3A5*3 carriers had a higher systemic exposure to atorvastatin and 2-OH atorvastatin than the CYP3A5*1/*1 carriers. Thus, *SLCO1B1* c.521T>C and CYP3A5*3 polymorphisms affect the pharmacokinetics of atorvastatin and 2-OH atorvastatin.

## 1. Introduction

Atorvastatin is a 3-hydroxy-3-methylglutaryl-coenzyme A reductase inhibitor, which is used for managing dyslipidemia [1,2]. Statins reduce cardiovascular morbidity and mortality in high-risk patients, and their efficacy and safety in facilitating the primary and secondary prevention of cardiovascular events have been demonstrated in various clinical trials [2].

However, individual responses to statins can vary considerably and may yield various adverse events. For example, statins can cause myopathy and even rhabdomyolysis, which is a rare adverse effect in certain patients [1,3,4]. This toxicity is often associated with increased plasma concentrations of statins caused by drug interactions or hereditary differences in statin pharmacokinetics, and this includes the effect of interindividual variability of enzymatic (e.g., CYP3A5) and transporter (e.g., SLCO1B1) activities caused by SNPs responsible for atorvastatin [3,5,6].

Atorvastatin is subject to an extensive first-pass metabolism in the gut wall and liver, with an oral bioavailability of 14% [7]. Recently, atorvastatin was identified as a substrate of SLCO1B1 (i.e., organic anion transporting polypeptide 1B1, OATP1B1) [8,9,10]. In addition, several single nucleotide polymorphisms (SNPs) in the gene encoding SLCO1B1 have been identified, and some, specifically the c.521T>C polymorphism, are associated with a reduced activity of SLCO1B1 and markedly elevated plasma levels of atorvastatin in vivo in humans [8,9,10]. The *SLCO1B1* c.388A>G polymorphism also affects the SLCO1B1 transporter function [11,12]. 

In hepatic metabolism, atorvastatin is hydroxylated primarily by CYP3A4 and CYP3A5, as well as by CYP2C8 at a low rate; this hydroxylation yields its major active metabolites, including 2-hydroxy (2-OH) atorvastatin and 4-hydroxy-atorvastatin [7]. In the CYP3A family, CYP3A4 is generally considered the dominant form. However, recent data suggested the relative contribution of CYP3A5 to the total CYP3A pool may be much larger than was previously suspected, particularly in individuals with increased expression levels of CYP3A5 [13,14,15]. The CYP3A5 protein is expressed polymorphically, and several genetic variants, of which CYP3A5*3 is the single common allele for major ethnic groups, can reduce the expression level of *CYP3A5* [16,17]. The genetic variants thereby cause variation in the pharmacokinetics and pharmacodynamics of the substrate [16]. Several in vivo human datasets have shown that the CYP3A5*3 genotype remarkably affects the pharmacokinetics of CYP3A substrates [18,19]. In addition, the pharmacokinetic and lipid-lowering effects of statins are reportedly related to *CYP3A4/5* genotypes [20,21]. 

Based on the evidence that the respective *SLCO1B1* and *CYP3A5* genotypes may affect the pharmacokinetics of atorvastatin and its active metabolite, this study assessed the simultaneous pharmacogenetic effects of *SLCO1B1* and *CYP3A5* genetic polymorphisms on the pharmacokinetics of atorvastatin and its active metabolite 2-OH atorvastatin in humans. 

## 2. Materials and Methods

### 2.1. Participants

A total of 46 healthy male volunteers were enrolled from previous pharmacokinetic studies, and all participants were recruited from a Korean population. Detailed physical examinations, 12-lead electrocardiograms, vital parameters, and laboratory tests, including blood chemistry, hematology, and urine analyses, were performed to determine the health status of the volunteers. The exclusion criteria were as follows: history or evidence of hepatic, renal, gastrointestinal, or hematological pathologies, hepatitis B or C or human immunodeficiency virus infections, any other acute or chronic disease, and an allergy to any drug. The participants were not allowed to consume any drug two weeks before or during the study period. All procedures were performed in accordance with the Declaration of Helsinki and the Good Clinical Practice guidelines.

### 2.2. Study Design

The study protocol was approved by the Institutional Review Board of Anam Hospital, Korea University College of Medicine (Seoul, Korea). For pharmacokinetic data, we used data obtained from the previous pharmacokinetic studies. All study participants were admitted to the clinical trial center during the evening prior to the day of drug administration. After fasting overnight, they were given a single 80 mg oral dose of atorvastatin (Pfizer Korea Ltd., Seoul, Korea) with 240 mL of water. The selected dosage was a highest maintenance dose for commercially used atorvastatin [1]. Blood samples were collected immediately before drug administration (baseline) and then at 0.17, 0.33, 0.5, 0.67, 0.83, 1, 1.25, 1.5, 1.75, 2, 2.5, 3, 4, 5, 6, 8, 10, 12, 24, 36, and 48 h after drug administration. Blood samples were collected in ethylenediaminetetraacetic acid (EDTA) tubes (Vacutainer, Becton Dickinson, Franklin Lakes, NJ, USA) and centrifuged at 1977 g and 4 °C for 15 min. Plasma samples were stored at −70 °C until analysis.

### 2.3. SLCO1B1 and CYP3A5 Genotyping 

For the genetic analysis, a blood sample was drawn from each individual and stored in EDTA at −20 °C until DNA extraction. DNA was extracted using standard methods (QIAamp DNA Blood Mini Kit; Qiagen, Hilden, Germany) [22,23]. All participants were genotyped for the SLCO1B1*1b (c.388A>G, rs2306283), SLCO1B1*5 (c.521T>C, rs4149056), and CYP3A5*3 (6986A > G, rs776746) alleles using previously described pyrosequencing methods [24,25]. The validity of this method was confirmed using direct sequencing.

### 2.4. Bioanalysis

The atorvastatin and 2-hydroxy (2-OH) atorvastatin concentrations were measured as described in a previous study, with a slight modification [26]. Briefly, the sample was injected into a high-performance liquid chromatography system (Shiseido Co., Ltd., Tokyo, Japan) coupled with an API 4000 mass spectrometer (Applied Biosystems-SCIEX, Framingham, MA, USA) equipped with a Capcell Pak C_18_ column (2.0 mm × 150 mm, 5 μm, Tokyo, Japan). The isocratic mobile phase was a mixture of acetonitrile (25%), methanol (40%), and 0.01% formic acid in water (35%). The flow rate of the mobile phase was 0.2 mL/min. The mass spectrometer was equipped with an electrospray ionization source and operated in negative ion mode with multiple reaction monitoring. The mass transition ion pairs of atorvastatin, 2-OH atorvastatin, and atorvastatin-d5 (internal standard) were selected as *m/z* 557.4 → 278.1, *m/z* 573.5 → 278.1, and *m/z* 573.5 → 278.1, respectively. Standard working solutions of atorvastatin and 2-OH atorvastatin (0.5, 1, 2, 5, 10, 20, 50, 100, and 200 ng/mL) were prepared by diluting the stock solution with blank plasma. Linear calibration curves of standard atorvastatin and 2-OH atorvastatin were established (*r*^2^ = 0.999). 

### 2.5. Pharmacokinetic Analysis

The pharmacokinetic variables of atorvastatin and 2-OH atorvastatin were estimated using non-compartmental methods utilizing WinNonlin version 7.0 (Pharsight, Cary, NC, USA) [27]. The peak concentrations (C_max_) and time to reach C_max_ (T_max_) were estimated directly from the observed plasma concentration–time data. AUC_last_ was calculated using the linear trapezoidal rule. The AUC from 0 to infinity (AUC_inf_) was calculated as AUC_inf_ = AUC_last_ + C_t_/K_e_ (where C_t_ is the last plasma concentration measured). The elimination rate constant (K_e_) was determined via linear regression analysis of the log-linear part of the plasma concentration–time curve. The half-life (t_1/2_) was calculated using the equation half-life = ln2/K_e_. Oral clearance (CL/F) of atorvastatin was calculated as follows: CL/F = dose/AUC_inf_.

### 2.6. Statistical Analysis

Data are expressed as mean ± standard deviation (SD) in the text and tables and, for clarity, as mean ± standard error of measurement (SEM) in the figures. Differences were considered significant at *p* < 0.05. The pharmacokinetic parameters of the *SLCO1B1* and *CYP3A5* genotypes were comparatively analyzed using one-way analysis of variance or the Kruskal–Wallis test, followed by Tukey’s post hoc analysis after examining the normal distribution of the data. Regarding the *SLCO1B1* c.521T>C polymorphism, only one participant with c.521CC was found, so pharmacokinetic comparisons were performed between participants with c.521TT and participants with c.521TC or c.521CC using an unpaired *t*-test. Genetic equilibrium and linkage disequilibrium were determined according to the Hardy–Weinberg equation using SNPalyzer version 7.0 (DYNACOM Co., Ltd., Yokohama, Japan). All statistical analyses were performed using the SAS statistical software package version 9.4. (SAS Institute, Cary, NC, USA).

## 3. Results

### 3.1. Demographics and Genotyping

The *SLCO1B1* and *CYP3A5* genotypes and their allele frequencies are presented in Table 1. 

Their demographic data according to the *SLCO1B1* and *CYP3A5* genotypes are presented in Table S3. 

CYP3A4*18 allele was also screened in this population, but no individuals were found with this allele. All observed genotype frequencies for *SLCO1B1* and *CYP3A5* were in the Hardy–Weinberg equilibrium. The observed allele frequencies for CYP3A5*3, *SLCO1B1* c.521T>C, and *SLCO1B1* c.388A>G in this population were 69.6%, 15.2%, and 71.7%, respectively. The genotype groups were compared based on an analysis of covariance utilizing an effective term for both *SLCO1B1* and *CYP3A5* genotypes as well as demographic data, including age, body weight, and height as covariates. However, the interactions between genotype and each of the covariates were not statistically significant. 

### 3.2. Effects of Polymorphic SLCO1B1 and CYP3A5 Genotypes

**Figure 1:**
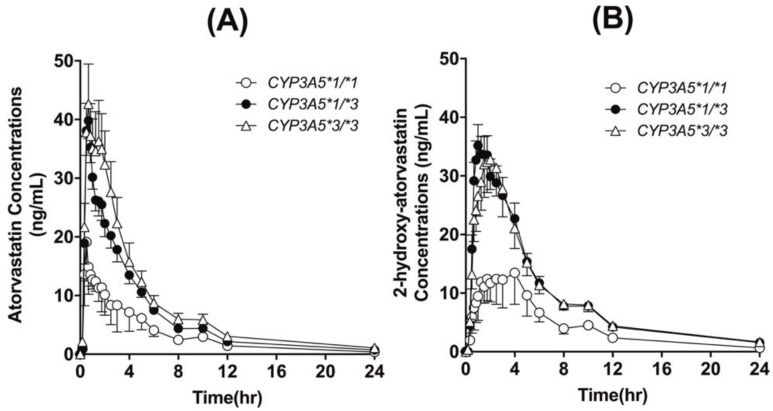
Mean (±SEM) plasma concentration-time curves of atorvastatin (**A**) and 2-OH atorvastatin (**B**) following oral administration of 80 mg atorvastatin, classified by *CYP3A5* genotype.

Individuals with CYP3A5*3 polymorphism exhibited elevated plasma concentration profiles of atorvastatin and 2-OH atorvastatin compared to those with CYP3A5*1/*1 (Figure 1). 

Additionally, the pharmacokinetic parameters of atorvastatin and its active metabolite, 2-OH atorvastatin, were significantly different between the *CYP3A5* genotype groups. The C_max_ and AUC_inf_ values of atorvastatin in the CYP3A5*3/*3 carriers were 2.6- and 2.8-fold higher, respectively, than those with CYP3A5*1/*1 carriers (*p* < 0.05). Regarding 2-OH atorvastatin pharmacokinetics, the AUC_inf_ values of CYP3A5*3/*3 carriers were 2.4-fold higher than those of CYP3A5*1/*1 carriers (*p* = 0.040); however, the C_max_ values were higher in CYP3A5*3 carriers, without a significant difference (*p* = 0.097, Table 2).

**Figure 2:**
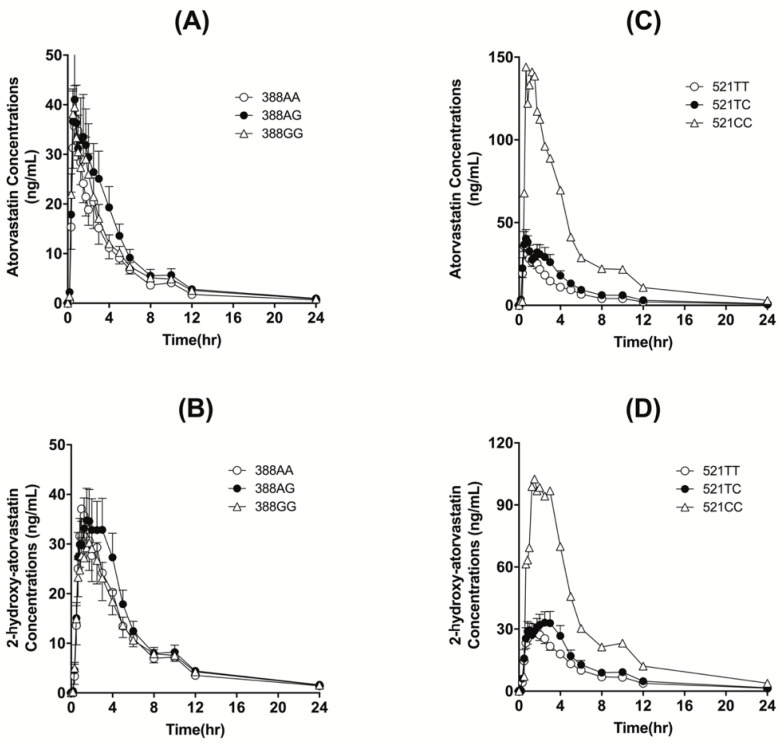
Mean (±SEM) plasma concentration–time curves of atorvastatin (**A**,**C**) and 2-OH atorvastatin (**B**,**D**) following oral administration of 80 mg atorvastatin, classified by *SLCO1B1* polymorphisms.

The *SLCO1B1* c.388A>G genotype did not influence the plasma concentration profiles of atorvastatin and 2-OH atorvastatin. However, *SLCO1B1* c.521T>C polymorphism affected the plasma concentration profiles in that the c.521CC carrier showed higher plasma concentration profiles for both atorvastatin and 2-OH atorvastatin than the c.521TT or c.521TC carriers (Figure 2). 

The pharmacokinetics of atorvastatin and 2-OH atorvastatin did not differ between the c.388A>G genotype groups (*p* > 0.05, Table 3). 

However, *SLCO1B1* c.521T>C substantially influenced the systemic exposures of atorvastatin and 2-OH atorvastatin: 150.27 ng·h/mL for c.521TT, 208.26 ng·h/mL for c.521TC, and 704.74 ng·h/mL for c.521CC for atorvastatin AUC_inf_ (*p* = 0.003), and 209.70 ng·h/mL for c.521TT, 259.91 ng·h/mL for c.521TC, and 673.98 ng·h/mL for c.521CC for 2-OH atorvastatin AUC_inf_ (*p* = 0.024, Table 4).

When AUC ratio of 2-OH atorvastatin to atorvastatin according to *SLCO1B1* polymorphisms were compared, neither *SLCO1B1* c.388A>G nor c.512C>T affected the ratio. Regarding CYP3A5*3 polymorphism, the AUC ratio decreased in a gene-dose-dependent manner (*p* = 0.049, Table 2).

### 3.3. Simultaneous Effects of SLCO1B1 c.521T>C and CYP3A5*3 Polymorphisms

**Figure 3:**
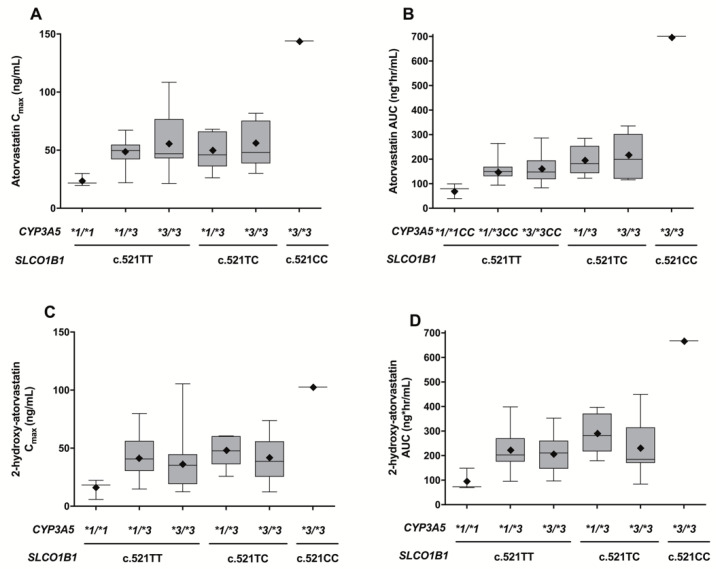
Box-and-whiskers plot of pharmacokinetic parameters following oral administration of 80 mg atorvastatin, grouped by *SLCO1B1* c.521T>C and CYP3A5*3 genotypes. (**A**,**B**) atorvastatin, (**C**,**D**) 2-OH atorvastatin. The horizontal lines with solid circles within each box represent the median. The box edges show the lower (25th) and upper (75th) quartiles. The whiskers extend from the 5th and 95th quartiles. The diamonds in solid boxes indicate the mean values.

Because both *SLCO1B1* c.521T>C and CYP3A5*3 substantially influenced the pharmacokinetics of atorvastatin and 2-OH atorvastatin, the simultaneous effects of both genotypes on their pharmacokinetics were assessed. As expected, *SLCO1B1* c.521TT/CYP3A5*1/*1 carriers showed lower C_max_ and AUC_inf_ values for atorvastatin and 2-OH atorvastatin than individuals with the *SLCO1B1* c.521T>C and/or CYP3A5*3 genotypes (*p* < 0.0001, Figure 3). 

In individuals with the *SLCO1B1* c.521TT genotype, the CYP3A5*3 carriers (155.9 ng·h/mL for atorvastatin, 215.3 ng·h/mL for 2-OH atorvastatin) showed a higher systemic exposure for atorvastatin and 2-OH atorvastatin than CYP3A5*1/*1 carriers (*p* = 0.0176) (72.8 ng·h/mL for atorvastatin, 96.9 ng·h/mL for 2-OH atorvastatin); however, the CYP3A5*1/*3 and *3/*3 carriers both exhibited comparable values (*p* > 0.05, Figure 3). Similarly, CYP3A5*3/*3 carriers (214.8 ng·h/mL for atorvastatin, 231.3 ng·h/mL for 2-OH atorvastatin) exhibited comparable AUC values with CYP3A5*1/*3 carriers (195.3 ng·h/mL for atorvastatin, 231.3 ng·h/mL for 2-OH atorvastatin) when compared within the *SLCO1B1* c.521TC carriers (*p* > 0.05). 

## 4. Discussion

This study demonstrated that both *SLCO1B1* c.521T>C and CYP3A5*3 genetic polymorphisms considerably influenced the pharmacokinetic variability of atorvastatin and its active metabolite, 2-OH atorvastatin. 

The SLCO1B1 transporter is mainly expressed on the sinusoidal membrane of hepatocytes and acts as an efflux pump for its substrates, including several HMG-CoA reductase inhibitors, angiotensin-converting enzyme (ACE) inhibitors, and angiotensin II receptor antagonists [28]. Atorvastatin is also a widely known substrate of SLCO1B1, and several previous studies have revealed that systemic exposure to atorvastatin is affected by *SLCO1B1* genetic polymorphisms [29]. In this study, atorvastatin pharmacokinetics were influenced by c.521T>C but not by c.388A>G. Although 2-OH atorvastatin has not yet been confirmed as a substrate of SLCO1B1, the possibility was suggested based on an observation of its increased disposition affected by the co-incubation of rifampin, an inhibitor of SLCO1B1 transporter, in an in vitro drug–drug interaction study [30]. *SLCO1B1* c.521T>C (but not c.388A>G) polymorphism affected the pharmacokinetics of 2-OH atorvastatin (like atorvastatin) in this study, and c.521T>C dependently increased its systemic exposure gene dose. Assuming that the AUC ratio of 2-OH atorvastatin/atorvastatin did not differ between the *SLCO1B1* genotype groups, this study suggest that 2-OH atorvastatin may be a substrate of the SLCO1B1 transporter. 

No significant difference in atorvastatin and 2-OH atorvastatin pharmacokinetics associated with the *SLCO1B1* c.388A>G polymorphism was observed. Although in vitro pharmacokinetic studies have suggested that this polymorphism may alter transporter functions [31,32], the results of clinical studies that have evaluated the effects of *SLCO1B1* c.388A>G on atorvastatin responses are conflicting. Some studies have shown a correlation between an improvement in the low-density lipoprotein (LDL) cholesterol-lowering effect of atorvastatin and the *SLCO1B1* c.388A>G polymorphism [12,32]. However, a recent study found no association between the LDL-lowering effect and *SLCO1B1* c.388A>G [11]. Notably, these studies did not evaluate the pharmacokinetics of atorvastatin and its metabolites, and until recently, there were no studies that evaluated the effect of *SLCO1B1* c.388A>G on atorvastatin and 2-OH atorvastatin pharmacokinetics. 

In addition to *SLCO1B1* genotypes, the CYP3A5*3 polymorphism substantially influenced the pharmacokinetics of atorvastatin and 2-OH atorvastatin in this study. Like the *SLCO1B1* c.521T>C polymorphism, the CYP3A5*3 polymorphism increased the systemic exposure of atorvastatin as well as that of 2-OH atorvastatin in a dose-dependent manner. CYP3A4/5 facilitates the metabolism of atorvastatin in 2-OH atorvastatin [33]. The AUC ratio of 2-OH atorvastatin/atorvastatin was consistently affected by the CYP3A5*3 polymorphism, thereby suggesting the involvement of CYP3A5 in the metabolism of atorvastatin in 2-OH atorvastatin. Researchers have recently reported that CYP3A5*3 polymorphism is a significant factor influencing the pharmacokinetic variability of atorvastatin [6]. Interestingly, their study showed that the CYP3A5*3 polymorphism reduced the systemic exposure of atorvastatin, contrary to the results of the present study. This study cannot fully explain the reasons behind the data of this study showing conflicting results with other studies. Several studies have shown that CYP3A5*3 polymorphism increases the intensity of atorvastatin-induced lipid-lowering effects and the risk of adverse events [4,21]. Assuming that the blood levels of atorvastatin are directly linked to its effects and adverse event risks, the findings are arguable. However, there are several confounding factors through which the CYP3A5*3 polymorphism may impact the disposition of atorvastatin. This study only recruited healthy male Korean participants to exclude some confounding factors for pharmacogenetic study, but there are several confounding factors (e.g., gender, the usage or lack of ezetimibe as a comedication, and ethnicity) that influence the variability of atorvastatin pharmacokinetics, and their effects could not be excluded [6]. Additional investigations are needed to clarify the discrepancies in these findings.

In addition to atorvastatin, CYP3A5*3 polymorphism increased the systemic exposure to 2-OH atorvastatin. Considering that CYP3A5 facilitates the metabolism of atorvastatin into 2-OH atorvastatin, it is appropriate that the systemic exposure of 2-OH atorvastatin decreases in tandem with the dysfunctional CYP3A5 enzymes via CYP3A5*3. However, the results showed that the CYP3A5*3 polymorphism increased the systemic exposure to 2-OH atorvastatin and atorvastatin. The half-life of 2-OH atorvastatin appeared to be longer in CYP3A5*3 carriers than in CYP3A5*1/*1 carriers, but the difference was not statistically significant. Therefore, CYP3A5 is likely not the main factor influencing the disposition of 2-OH atorvastatin. Being formed from atorvastatin, 2-OH atorvastatin is further metabolized into 2-OH atorvastatin lactone via uridine diphosphate–glucuronosyltransferase (UGT) 1A1 and UGT1A3 [3,7]. Considering that lactone formation is not affected by CYP3A5, an alternative mechanism through which the CYP3A5*3 polymorphism may alter the disposition of 2-OH atorvastatin may be the difference in the binding affinity of both atorvastatin types to the SLCO1B1 transporter. A drug interaction study showed that gemfibrozil substantially and simultaneously increased both atorvastatin and 2-OH atorvastatin blood levels [34]. Gemfibrozil is a potent inhibitor of CYP2C8, but not CYP3A4/5 [35,36]. It is also a potent inhibitor of the SLCO1B1 transporter, so both the atorvastatin and 2-OH atorvastatin levels are increased via the inhibition of said transporter [34]. Similarly, cyclosporine, a potent inhibitor of the SLCO1B1 transporter, has also increased the blood levels of both atorvastatin types [37,38]. The present study has consistently demonstrated the increased systemic exposure of atorvastatin and 2-OH atorvastatin caused by the *SLCO1B1* c.521T>C polymorphism. Assuming that the binding affinities of atorvastatin and 2-OH atorvastatin to the SLCO1B1 transporter are different, the elimination of 2-OH atorvastatin may be reduced and preserved longer despite the relatively reduced formation of 2-OH atorvastatin caused by the CYP3A5*3 polymorphism. When the cellular uptake of atorvastatin was measured in the absence and presence of 2-OH atorvastatin in HEK293 cells, the atorvastatin uptake was inhibited and reduced by approximately 65% and 15%, respectively, by 2-OH atorvastatin at concentrations of 1 and 10 μM, respectively [30]. These results suggest that atorvastatin and 2-OH atorvastatin act competitively on transporters, and that their permeabilities are different.

This study had some limitations. First, it did not measure the lactone form of the 2-OH atorvastatin, which may provide another clue in evaluating the role of CYP3A5*3 in atorvastatin metabolism. Hydroxylated metabolites reportedly demonstrate approximately 70% of the pharmacological activity of atorvastatin, but the lactone form is pharmacologically inactive [3,7]. Second, actual pharmacodynamic effects were not compared with the exposure of the active metabolites, as this was a single-dose treatment study. However, the simultaneously increased blood levels of atorvastatin and 2-OH atorvastatin may exhibit an additive or synergistic effect on their lipid-lowering intensities and/or the incidence rate of statin-induced adverse events. Lastly, there was only one person with genotype CYP3A5*3/*3 with *SLCO1B1* c.521TT. Further and larger-scale clinical research is necessary to confirm the combined influence of the genotypes.

## 5. Conclusions

The results of this study suggest that both *SLCO1B1* c.521T>C and CYP3A5*3 polymorphisms influence the pharmacokinetics of atorvastatin and its active metabolite, 2-OH atorvastatin. In other words, presumably, the use of statin in the patients with *SLCO1B1 c.521T>C* and CYP3A5*3 polymorphisms may be more effective or may also yield adverse events based on the results in this study, considering the effect of SNPs on the atorvastatin pharmacokinetics. 

## Figures and Tables

**Table 1:** Frequencies of *SLCO1B1* and *CYP3A5* genetic variations evaluated in this study.

	Genotype	Frequency (%)	Allele	Frequency (%)
CYP3A5*3	*1/*1	3 (6.5%)	*1	30.4%
(rs776746)	*1/*3	22 (47.8%)	*3	69.6%
	*3/*3	21 (45.7%)		
***SLCO1B1* c.521T>C**	TT	33 (71.7%)	T	84.8%
(rs4149056)	TC	12 (26.1%)	C	15.2%
	CC	1 (2.2%)		
***SLCO1B1* c.388A>G**	AA	6 (13.0%)	A	28.3%
(rs2306283)	AG	15 (30.4%)	G	71.7%
	GG	27 (56.5%)		

**Table 2:** Pharmacokinetic parameters of atorvastatin and 2-OH atorvastatin following administration of 80 mg atorvastatin, classified by CYP3A5*3 genotype.

CYP3A5 Genotype	CYP3A5*1/*1	CYP3A5*1/*3	CYP3A5*3/*3	*p*-Value
Individuals (*n*)	3	22	21	
Atorvastatin				
Half-life (h)	5.18 ± 2.42	7.87 ± 2.17	8.28 ± 1.74	0.052
T_max_ (h)	0.86 ± 0.77	0.94 ± 0.88	1.00 ± 0.61	0.483
C_max_ (ng/mL)	23.77 ± 5.50	49.04 ± 11.90	61.15 ± 27.99	0.012 ^a,^*
AUC_all_ (ng·h/mL)	72.78 ± 30.57	161.03 ± 47.85	209.74 ± 131.03	0.010 ^a,^*
AUC_inf_ (ng·h/mL)	75.86 ± 26.98	162.79 ± 49.62	211.56 ± 131.45	0.010 ^a,^*
CL/F (L/h)	1164.3 ± 470.4	532.2 ± 151.4	463.0 ± 173.6	<0.001 ^a,b,^*
2-OH atorvastatin				
Half-life (h)	7.47 ± 3.41	8.98 ± 1.90	9.39 ± 2.27	0.644
T_max_ (h)	2.02 ± 1.72	1.45 ± 0.89	1.50 ± 0.72	0.789
C_max_ (ng/mL)	15.50 ± 8.58	43.34 ± 16.66	42.71 ± 25.05	0.097
AUC_all_ (ng·h/mL)	96.911 ± 44.67	238.50 ± 85.82	244.80 ± 128.63	0.030 ^a,b,^*
AUC_inf_ (ng·h/mL)	109.39 ± 38.47	242.49 ± 88.65	249.22 ± 129.18	0.040 ^a,b,^*
AUC Ratio	1.54 ± 0.61	1.49 ± 0.39	1.21 ± 0.33	0.049 ^a,^*
(2-OH atorvastatin/atorvastatin)				

Data are expressed as mean ± SD. ^a^, *1/*1 vs. *3/*3; ^b^, *1/*1 vs. *1/*3, * *p* < 0.05. C_max_, peak plasma concentration; T_max_, time to C_max_; AUC_all_, area under the time versus concentration curve from 0 to 24 h; AUC_inf_, AUC from 0 to infinity.

**Table 3:** Pharmacokinetic parameters of atorvastatin and 2-OH atorvastatin following administration of 80 mg atorvastatin, classified by *SLCO1B1* c.388A>G polymorphisms.

	c.388AA	c.388AG	c.388GG	*p*-Value
Individuals (*n*)	6	14	26	
Atorvastatin				
Half-life (h)	0.09 ± 0.02	0.08 ± 0.01	0.10 ± 0.04	0.619
T_max_ (h)	7.74 ± 1.75	8.35 ± 1.73	7.67 ± 2.36	0.390
C_max_ (ng/mL)	0.819 ± 0.309	1.166 ± 1.090	0.839 ± 0.564	0.529
AUC_all_ (ng·h/mL)	42.751 ± 11.328	52.018 ± 31.393	54.716 ± 19.825	0.464
AUC_inf_ (ng·h/mL)	146.14 ± 39.051	201.82 ± 152.98	168.23 ± 73.396	0.458
CL/F (L/h)	147.32 ± 39.909	203.95 ± 153.58	170.14 ± 73.819	0.747
2-OH atorvastatin				
Half-life (h)	9.52 ± 1.49	9.65 ± 2.61	8.68 ± 2.03	0.358
T_max_ (h)	1.02 ± 0.18	1.79 ± 1.24	1.42 ± 0.68	0.174
C_max_ (ng/mL)	40.21 ± 18.94	44.07 ± 24.33	38.90 ± 21.67	0.782
AUC_all_ (ng·h/mL)	223.33 ± 96.387	256.59 ± 146.55	214.05 ± 94.209	0.526
AUC_inf_ (ng·h/mL)	227.24 ± 98.987	261.32 ± 146.88	218.89 ± 94.842	0.530
AUC Ratio	1.55 ± 0.71	1.34 ± 0.27	1.34 ± 0.36	0.482
(2-OH atorvastatin/atorvastatin)				

Data are expressed as mean ± SD. C_max_, peak plasma concentration; T_max_, time to C_max_; AUC_all_, area under the time versus concentration curve from 0 to 24 h; AUC_inf_, AUC from 0 to infinity.

**Table 4:** Pharmacokinetic parameters of atorvastatin and 2-OH atorvastatin following administration of 80 mg atorvastatin, classified by *SLCO1B1* c.521T>C polymorphisms.

	c.521TT	c.521TC	c.521CC	c.521TC or c.521CC	*p*-Value
Individuals (*n*)	33	12	1	13	
Atorvastatin					
Half-life (h)	7.84 ± 2.38	8.07 ± 1.20	7.31	8.01 ± 1.17	0.812
T_max_ (h)	0.86 ± 0.68	1.15 ± 0.90	0.66	1.12 ± 0.88	0.295
C_max_ (ng/mL)	49.16 ± 18.81	53.41 ± 18.78	144.0	60.39 ± 30.92	0.139
AUC_all_ (ng·h/mL)	148.35 ± 51.206	206.67 ± 78.036	700.80	244.68 ± 156.09	0.003 *
AUC_inf_ (ng·h/mL)	150.27 ± 52.008	208.26 ± 78.133	704.74	246.46 ± 156.70	0.003 *
CL/F (L/h)	607.22 ± 264.20	438.18 ± 163.14	113.51	413.21 ± 180.30	0.019 *
2-OH atorvastatin					
Half-life (h)	9.18 ± 2.34	8.92 ± 1.80	7.90	8.85 ± 1.75	0.641
T_max_ (h)	1.41 ± 0.85	1.69 ± 0.97	1.5	1.68 ± 0.94	0.361
C_max_ (ng/mL)	37.47 ± 20.79	44.20 ± 17.39	102.5	48.70 ± 23.22	0.118
AUC_all_ (ng·h/mL)	204.57 ± 82.641	256.57 ± 106.30	667.80	288.21 ±152.86	0.02 *
AUC_inf_ (ng·h/mL)	209.70 ± 83.89	259.91 ± 106.47	673.98	291.77 ± 153.56	0.024 *
AUC Ratio	1.42 ± 0.41	1.26 ± 0.32	0.96	1.24 ± 0.41	0.172
(2-OH atorvastatin/atorvastatin)					

Data are expressed as mean ± SD, * *p* < 0.05. C_max_, peak plasma concentration; T_max_, time to C_max_; AU_Call_, area under the time versus concentration curve from 0 to 24 h; AUC_inf_, AUC from 0 to infinity.

## Data Availability

The data presented in this study are available upon reasonable request from the corresponding author. The data are not publicly available because of privacy concerns.

## Supplementary Materials

The following supporting information can be downloaded at: https://www.mdpi.com/article/10.3390/pharmaceutics14071491/s1, Table S1: Accuracy and precision of atorvastatin and 2-OH atorvastatin in plasma, Table S2: Stability of atorvastatin and 2-OH atorvastatin in plasma, Table S3: Demographic data, Figure S1: Representative chromatograms of 200 ng/mL of atorvastatin (a), 200 ng/mL of 2-OH atorvastatin (b), and 200 ng/mL of atorvastatin-d5 (c).
