# Supplementary material for: CYP3A5*3 and SLCO1B1 c.521T>C Polymorphisms Influence the Pharmacokinetics of Atorvastatin and 2-Hydroxy Atorvastatin
Source: Pharmaceutics. 2022 Jul 18;14(7):1491. doi: 10.3390/pharmaceutics14071491 (PMC9323915; doi:10.3390/pharmaceutics14071491)
Supplement: Supplementary file 1 [file pharmaceutics-14-01491-s001.zip › pharmaceutics-1793787-supplementary.pdf]

Table S1. Accuracy and precision of atorvastatin and 2-OH atorvastatin in plasma.

**Concentration (ng/mL)**	**Accuracy (%)**		**Precision (%)**	
	Intra-day	Inter-day	Intra-day	Inter-day
	Atorvastatin			
0.2 (LLOQ)	104.7	101.3	6.3	4.4
5	100.9	103.4	4.6	1.3
100	99.9	98.6	3.3	3.3
200	93.0	96.5	3.6	3.8
	2-OH atorvastatin			
0.2 (LLOQ)	104.7	100.5	3.4	5.4
5	97.2	101.3	4.7	2.5
100	100.9	100.4	2.8	3.1
200	96.3	98.6	3.9	2.5

LLOQ, lower limit of quantification.

Table S2. Stability of atorvastatin and 2-OH atorvastatin in plasma.

**Stability**	**Storage condition**	**Concentration (ng/mL)**	**Remained (%)**
Atorvastatin			
Short-Term	6 hr, room temperature	0.5	100.8 ± 4.8
		200	101.1 ± 5.8
Freeze and Thaw	4 cycles, −80 ˚C	0.5	104.2 ± 4.1
		200	102.8 ± 3.6
Autosampler	36 hr, 4 ˚C	0.5	102.2 ± 1.9
		200	96.5 ± 0.8
Long-Term	1 week, −80 ˚C	0.5	102.8 ± 5.6
		200	103.5 ± 5.2
2-OH atorvastatin			
Short-Term	6 hr, room temperature	0.5	97.3 ± 7.7
		200	102.0 ± 4.3
Freeze and Thaw	4 cycles, −80 ˚C	0.5	105.9 ± 6.1
		200	104.0 ± 9.1
Autosampler	36 hr, 4 ˚C	0.5	108.1 ± 0.4
		200	103.1 ± 1.7
Long-Term	1 week, −80 ˚C	0.5	100.9 ± 2.8
		200	99.8 ± 2.4

Data are expressed as mean ± SD.

Table S3. Demographic data.

	genotype	*n*	Age (years)	Height (cm)	Bodyweight (kg)	BMI (kg/m²)
CYP3A5	**1/*1*	3	26.0 ± 1.7	176.0 ± 5.2	71.3 ± 3.1	23.0 ± 0.9
	**1/*3*	22	23.9 ± 2.3	174.8 ± 3.8	70.9 ± 6.9	23.2 ± 1.8
	**3/*3*	21	24.2 ± 2.6	176.5 ± 4.6	70.7 ± 7.3	22.6 ± 1.6
SLCO1B1 c.388A>G	TT	6	23.7 ± 0.5	174.5 ± 4.2	66.5 ± 4.8	21.8 ± 1.5
	TC	14	23.2 ± 2.9	175.1 ± 4.3	71.4 ± 7	23.3 ± 1.9
	CC	26	24.8 ± 2.3	176.2 ± 4.3	71.5 ± 6.9	23.0 ± 1.5
SLCO1B1 c.521T>C	AA	1	23	176	67	21.6
	AG	12	23.1 ± 2.3	176.5 ± 4.7	71.8 ± 6.3	23.0 ± 1.4
	GG	33	24.6 ± 2.4	175.3 ± 4.2	70.6 ± 7.1	22.9 ± 1.8

Data are expressed as mean ± SD.

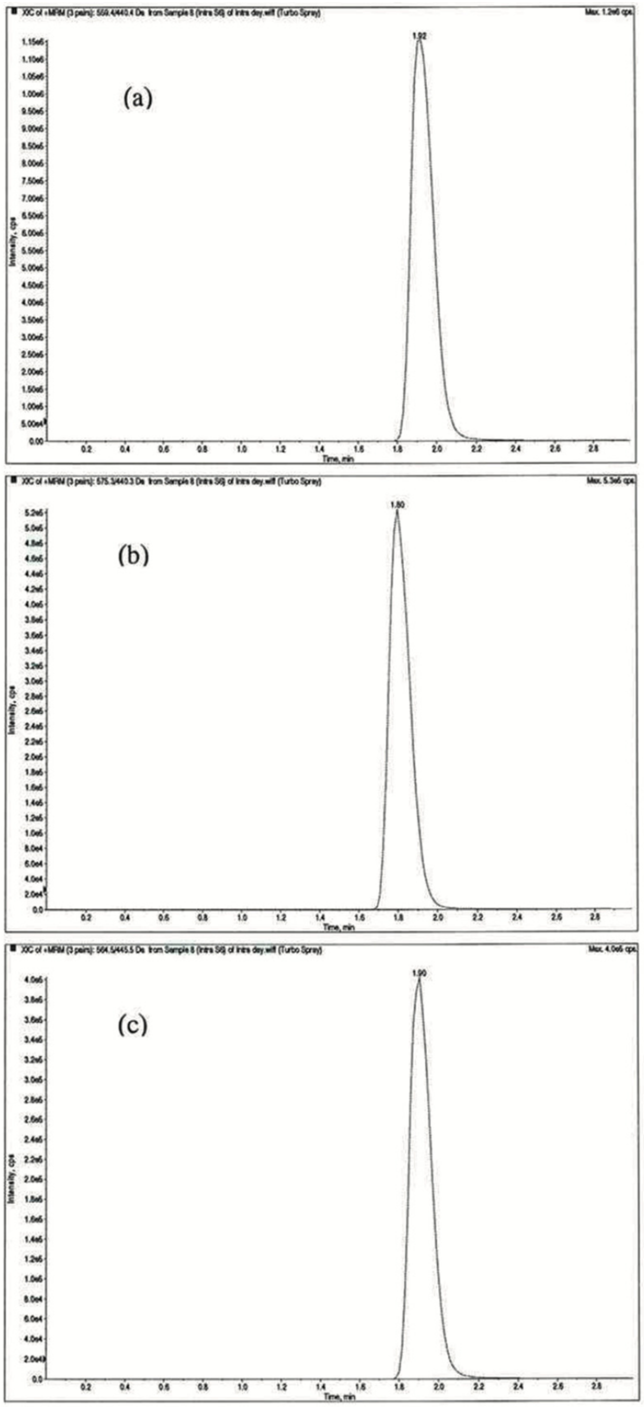

Figure S1. Representative chromatograms of 200 ng/mL of atorvastatin (a), 200 ng/mL of 2-OH atorvastatin (b), and 200 ng/mL of atorvastatin-d5 (c).
